# Trends of testis-sparing surgery for pediatric testicular tumors in South China

**DOI:** 10.1186/s12893-017-0230-1

**Published:** 2017-03-27

**Authors:** Yun-lin Ye, Qiu-ming He, Fu-fug Zheng, Sheng-jie Guo, Fang-jian Zhou, Zi-ke Qin

**Affiliations:** 1 Department of Urology, Sun Yat-sen University Cancer Center, State Key Laboratory of Oncology in South China, Collaborative Innovation Center for Cancer Medicine, Guangzhou, Guangdong 510060 China; 2 Department of Urology, The First Affiliated Hospital of Sun Yat-Sen University, Guangzhou, Guangdong 510080 China; 3 Department of Urology, Jiangxi Cancer Hospital, Nanchang, 330002 China

**Keywords:** Testicular tumor, Children, Benign tumor, Testis-sparing

## Abstract

**Background:**

Testis-sparing surgery is not popular in South China. This study aimed to investigate this procedure for pediatric testicular tumors.

**Methods:**

Children with testicular benign tumors were retrospectively analyzed from January 2001 to June 2015 in the Sun Yat-sen University Cancer Center (SYSUCC) and the First Affiliated Hospital (SYSU-1st). Follow-up was performed until June 2016, and the proportions of TSS in the two hospitals during the different periods were compared.

**Results:**

Forty-seven children with testicular benign tumors were enrolled, and 16 cases underwent testis-sparing surgery. All patients were cured and discharged, which included mature teratoma (*n* = 37), testicular adrenal rest tumors (*n* = 4), epidermal cysts (*n* = 3), granulomatous inflammation (*n* = 2) and adenomatoid tumors (*n* = 1). Inguinal testis-sparing surgery was performed in 16 children, and no recurrence was detected during follow-up. It was performed more frequently in SYSUCC than in SYSU-1st (*P* = 0.031), and the tumor size of these patients was smaller than those of patients who underwent radical orchiectomy (*P* = 0.044). Moreover, testis-sparing surgery has become more common in the past 5 years, although differences over time have not reached significance (*P* = 0.051).

**Conclusions:**

Testis-sparing surgery is reliable, and tumor size and special hospitals affect its success. Additionally, its use has become more popular in recent years. However, advocacy is still needed for the use of this technique in pediatric testicular benign tumors that are small sized.

**Electronic supplementary material:**

The online version of this article (doi:10.1186/s12893-017-0230-1) contains supplementary material, which is available to authorized users.

## Background

Testicular tumors are one of the most common solid tumors in children, but their incidence is as low as 0.5–2.0/100 000 [1–4]. Because of their rarity, they are often overlooked during clinical management and treated as to their adult counterparts [5, 6]. Recent studies have found that there are significant differences between testicular tumors in children and adults [7–9]. Especially, the proportion of benign testicular tumors in children is significantly higher than that in adults, which should affect to the evolution of the diagnosis and treatment for pediatric testicular tumors [2, 10, 11]. In recent years, it has been reported that testis-sparing surgery (TSS) could control pediatric testicular benign tumors effectively and retain the normal tissue of the testis [12–15]. However, the role of TSS is controversial, and indications and feasibility are uncertain. In this study, a retrospective analysis was performed to investigate the outcome and trend of TSS for testicular benign tumors of children in two high-volume centers.

The Sun Yat-sen University Cancer Center (SYSUCC) and the First Affiliated Hospital (SYSU-1st) are two high-volume medical centers in South China. Due to their high standing status, this report might be a fair representation of pediatric testicular benign tumors in South China.

## Methods

The data of patients with testicular tumors from January 2001 to June 2015 were retrospectively analyzed. Patients aged >12 years and those with non-primary lesions were excluded. The pathology reports were retrieved by an experienced pathologist (Liao), and benign tumors were screened. Clinical data including age, presentation, serum markers (alpha fetoprotein, AFP; human chorionic gonadotropin, HCG; lactate dehydrogenase, LDH), images, management and follow-up were recorded.

For boys with normal serum markers or other characters of a benign tumor, testis-sparing surgery was taken into consideration. With informed consent, after dissecting the tumor en bloc through an inguinal incision, an intra-operative biopsy was performed. If no malignant tumor was detected, testis-sparing surgery was performed, or a radical orchiectomy was performed.

Close follow-up was required for all patients every 3–6 months for 1 year and then annually, including the general condition, growth and development, recent physical examination, and scrotum and/or retroperitoneal ultrasonography.

Using the SPSS 21.0 Package, the proportions of testis-sparing surgery in the two hospitals during different periods were compared with a *χ* ^2^ test and rank sum test. *P* < 0.05 was considered to be significant.

## Results

From January 2001 to June 2015, a total of 47 children with benign testicular tumors were treated in the two hospitals, including 16 cases that underwent testis-sparing surgery. The patients were aged from 3 months to 141 months, with a median age of 38 months. There were 21 cases on the left side, 22 cases on the right side, and 4 cases that were bilateral; the latter were all testicular adrenal rest tumors. The maximum diameter of tumors ranged from 0.3 cm to 5.4 cm, and the median size was 2.5 cm. The preoperative level of serum AFP was 9.30–138.2 μg/L (normal range: 0.00–25.00 μg/L), and 6 patients aged 4–14 months had elevated AFP, with a median level of 56.0 μg/L (range from 30.3–138.2 μg/L). Elevated levels of HCG and LDH were not detected.

The numbers of mature teratomas, testicular adrenal rest tumors, epidermal cysts, granulomatous inflammation and adenomatoid tumors were 37, 4, 3, 2 and 1, respectively, and all patients were cured and discharged. Inguinal testis-sparing surgery was performed on 16 children, including 8 with mature teratomas, 4 with testicular adrenal rest tumors, 3 with epidermal cysts and 1 with an adenomatoid tumor. Radical orchiectomy was performed in 27 children who were referred to these two centers, and the other 4 orchiectomies were performed in basic hospitals, including 2 who received a scrotal orchiectomy (comparisons between the two groups are shown in Table 1). Of these 31 patients, 29 had teratoma and 2 had granulomatous inflammation.

**Table 1:** Characteristics of children underwent Testis-sparing surgery and Radical orchiectomy

	TSS (16)	RO (31)	*P*
Age (years)	5.5 (0.3–12)	2.1 (0.4–12)	0.238
Size (cm)	1.6 (0.3–2.4)	2.7 (1.3–8.0)	0.044
AFP (elevated proportion)	1/16	5/31	0.648
Period			
(after 2010 before 2010)	11 5	12 19	0.051
Hospital			
(SYSUCC: SYSU-1st)	12 4	13 18	0.031

*TSS* testis-sparing surgery

*RO* radical orchiectomy

During the operation, 21 frozen section examinations (FSEs) were performed, and 18 were benign disease. Sixteen patients underwent testis-sparing surgery, and the other two were suspended for mini-residual normal tissues (<1.0 cm). For patients who underwent testis-sparing surgery, the maximum diameter of the tumors ranged from 0.3 cm to 2.4 cm, and the median size was 1.6 cm, which was smaller than in those who underwent orchiectomy (*P* = 0.044). One child with an epidermal cyst had an AFP level of 76.2 μg/L. Only 4 children with testicular adrenal rest tumors and 1 with a mature teratoma underwent TSS before 2010 (24 children in all), which was lower than in the most recent 5 years (*P* = 0.051). The proportion of TSS was 12/25 in the cancer center, which was higher than the proportion in the general hospital (12/25 vs. 4/22, *P* = 0.031) (as shown in Table 1).

Until June 2016, only 28 cases were still actively followed in the hospital, including 15 patients who underwent TSS, and the remaining patients were followed up with by telephone. One patient was lost after follow up of 7 months. The median follow-up time was 56 months, with a range from 11 months to 147 months. No patient had complications, such as tumor recurrence, metastasis or testicular atrophy, and there were 3 cases of natural fertility.

## Discussion

During January 2001 to June 2015, 171 children with testicular tumors were treated in our two affiliated hospitals, and the proportions of benign tumors and teratoma were 27% and 22%, respectively, which were lower than the proportions reported in Europe and the United States (74% and 48%, respectively) but close to those in South Korea (47% and 39%, respectively) [2, 4, 10], which might be due to geographical factors. For their benign biological behavior, testis-sparing surgery was performed in these patients. Initially, testicular benign tumors with a size of 2–3 cm were a candidate for TSS, so preoperative evaluation was the basis for this procedure [16, 17].

Testicular tumor markers and ultrasound are two important tools in the evaluation of pediatric testicular tumors. Because yolk sac tumors are the most common malignant germ cell tumors in children, and AFP is elevated in >90% of pediatric yolk sac tumors, it is important to note that serum AFP levels in infants are normally higher than those in adults and reduce to normal levels by the age of 1. Therefore, for children <1 year old with testicular tumors, an elevated AFP level could be detected in those with benign tumors. On the other hand, for children >1 year, a normal level of AFP often indicates a benign tumor. Additionally, ultrasound could help distinguish the tumor origin and type, and it can play a role in evaluating normal tissues for testis-sparing candidates [18]. In this study, elevated AFP was detected in 6 children, and 5 of them underwent radical orchiectomy, while the other one had typical ultrasound characteristics of an epidermal cyst. Although boys with an elevated AFP level were inclined to have a malignant tumor and undergo radical orchiectomy, no significance was detected in this study. Therefore, for boys less than 1 year old, an elevated level of AFP was not indicative of a malignant tumor. Combined with well circumscribed ultrasound characteristics and decreased blood flow, a benign tumor should be taken into consideration. Additionally, during the operation, ultrasound could help to mark the tumor location when it was nonpalpable [19]. Other imaging, such as CT or MRI, was suggested to determine the stage when suspicion of malignancy was presented [20].

Generally, testicular tumors with a size <2.5 cm in diameter are defined as small testicular masses, and testis-sparing surgery has increasingly been performed for these lesions [21]. Additionally, masses with a diameter <2 cm were considered to be good candidates for TSS because nearly 80% of these cases were benign [22, 23]. In this study, tumor size ranged from 0.3 cm to 2.4 cm, and 2 cases of TSS were suspended due to scant residual normal tissues. The size of the TSS group was smaller than that of the radical orchiectomy group, and this might be related to the smaller size associated with a high proportion of benign tumors and difficulty of saving normal testicular tissues in larger size tumors.

Testis-sparing surgery is becoming increasingly more common for the treatment of pediatric testicular tumors, and long-term outcomes are favorable, although the number of reported cases is still small (11–21 cases) [14, 19, 24, 25]. For preoperative evaluation, including ultrasonography and serum markers, an FSE was critical to this procedure, and tumors with benign characteristics and size <2 cm were indicated. In this study, detailed examination and consultation were performed preoperatively, and an FSE was performed by dedicated pathologists. Testis-sparing surgery was more common in SYSUCC, which might be due to the unpopularity of testis-sparing surgery in South China. Although testis-sparing surgery has been performed more frequently in recent years, this was not statistically significant. Few testis-sparing surgeries were accomplished in general hospitals, including SYSU-1st and other basic hospitals. Maybe this was because it was not totally accepted by the urologists.

At present, a combination of preoperative evaluation and intra-operative biopsy could help urologists distinguish testicular benign tumors and malignant tumors effectively [22]. In fact, it was feasible and reliable in this study as well as in recent reports, and no recurrence or atrophy was recorded [5, 25]. Moreover, preservation of the testis could reduce the physiological and psychological impact on patients [24]. Therefore, a careful preoperative evaluation of pediatric testicular tumors, including scrotal ultrasound and serum markers, should be taken into consideration, and testis-sparing surgery should be offered to children with benign tumors if possible. In our opinion, the tumor size (<2.5 cm) and the volume of residual normal tissues (>1.0 cm) were important indicators for this procedure.

Though our series was not large and it was retrospectively analyzed, it presents a profile of testis-sparing surgery in South China, and we suggest and advocate testis-sparing surgery for pediatric testicular benign tumors.

## Conclusions

Benign testicular tumors in children are not rare, and testis-sparing surgery is reliable. Tumor size and special hospitals affect the success of TSS and it should be advocated for the treatment of pediatric testicular tumors.

## Additional file

Additional file 1: Clinical characters of children with testicular benign tumors. (PDF 24 kb)

## Abbreviations

AFP: Alpha fetoprotein
HCG: Human chorionic gonadotropin
LDH: Lactate dehydrogenase
SYSU-1st: The First Affiliated Hospital of Sun Yat-sen University
SYSUCC: Sun Yat-sen University Cancer Center
TSS: Testis-sparing surgery

## References

[1] Walsh TJ, Grady RW, Porter MP, Lin DW, Weiss NS (2000). Incidence of testicular germ cell cancers in U.S. children: SEER program experience 1973 to. Urology.

[2] Lee SD (2004). Epidemiological and clinical behavior of prepubertal testicular tumors in Korea. J Urol.

[3] Kaplan GW, Cromie WC, Kelalis PP, Silber I, Tank ES (1988). Prepubertal yolk sac testicular tumors--report of the testicular tumor registry. J Urol.

[4] Brosman SA (1979). Testicular tumors in prepubertal children. Urology.

[5] Ross JH (2009). Prepubertal testicular tumors. Urology.

[6] Treiyer A, Blanc G, Stark E, Haben B, Treiyer E, Steffens J (2007). Prepubertal testicular tumors: frequently overlooked. J Pediatr Urol.

[7] Ye YL, Qin ZK, Zhou FJ, Han H, Liu ZW, Yu SL, Li YH, Chen ZF (2008). Clinical analysis of stage I pediatric testicular yolk sac tumors: a report of ten cases. Ai Zheng.

[8] Schneider DT, Calaminus G, Koch S, Teske C, Schmidt P, Haas RJ, Harms D, Gobel U (2004). Epidemiologic analysis of 1,442 children and adolescents registered in the German germ cell tumor protocols. Pediatr Blood Cancer.

[9] Carver BS, Al-Ahmadie H, Sheinfeld J (2007). Adult and pediatric testicular teratoma. Urol Clin North Am.

[10] Ye YL, Sun XZ, Zheng FF, Bian J, Huang YP, Zhang XQ, Li ZX, Nie Y, Qin ZK, Dai YP (2012). Clinical analysis of management of pediatric testicular germ cell tumors. Urology.

[11] Pohl HG, Shukla AR, Metcalf PD, Cilento BG, Retik AB, Bagli DJ, Huff DS, Rushton HG (2004). Prepubertal testis tumors: actual prevalence rate of histological types. J Urol.

[12] Oottamasathien S, Thomas JC, Adams MC, DeMarco RT, Brock JW, Pope JC (2007). Testicular tumours in children: a single-institutional experience. BJU Int.

[13] Metcalfe PD, Farivar-Mohseni H, Farhat W, McLorie G, Khoury A, Bagli DJ (2003). Pediatric testicular tumors: contemporary incidence and efficacy of testicular preserving surgery. J Urol.

[14] Shukla AR, Woodard C, Carr MC, Huff DS, Canning DA, Zderic SA, Kolon TF, Snyder HM (2004). Experience with testis sparing surgery for testicular teratoma. J Urol.

[15] Xu X, Ye Y, Guo S, Zhou F, Han H, Liu Z, Qin Z (2014). Clinical analysis of pediatric testicular benign tumors. Nan Fang Yi Ke Da Xue Xue Bao.

[16] Pearse I, Glick RD, Abramson SJ, Gerald WR, Shamberger RC, La Quaglia MP (1999). Testicular-sparing surgery for benign testicular tumors. J Pediatr Surg.

[17] Rushton HG, Belman AB, Sesterhenn I, Patterson K, Mostofi FK (1990). Testicular sparing surgery for prepubertal teratoma of the testis: a clinical and pathological study. J Urol.

[18] Patel AS, Coley BD, Jayanthi VR (2007). Ultrasonography underestimates the volume of normal parenchyma in benign testicular masses. J Urol.

[19] Borghesi M, Brunocilla E, Schiavina R, Gentile G, Dababneh H, Della Mora L, Del Prete C, Franceschelli A, Colombo F, Martorana G (2015). Role of testis sparing surgery in the conservative management of small testicular masses: oncological and functional perspectives. Actas Urol Esp.

[20] Ahmed HU, Arya M, Muneer A, Mushtaq I, Sebire NJ (2010). Testicular and paratesticular tumours in the prepubertal population. Lancet Oncol.

[21] Sugita Y, Clarnette TD, Cooke-Yarborough C, Chow CW, Waters K, Hutson JM (1999). Testicular and paratesticular tumours in children: 30 years’ experience. Aust N Z J Surg.

[22] Woo LL, Ross JH (2016). The role of testis-sparing surgery in children and adolescents with testicular tumors. Urol Oncol.

[23] Bujons A, Sfulcini JC, Pascual M, Feu OA, Garat JM, Villavicencio H (2011). Prepubertal testicular tumours and efficacy of testicular preserving surgery. BJU Int.

[24] Ferretti L, Sargos P, Gross-Goupil M, Izard V, Wallerand H, Huyghe E, Rigot JM, Durand X, Benoit G, Ferriere JM (2014). Testicular-sparing surgery for bilateral or monorchide testicular tumours: a multicenter study of long-term oncological and functional results. BJU Int.

[25] Trobs RB, Krauss M, Geyer C, Tannapfel A, Korholz D, Hirsch W (2007). Surgery in infants and children with testicular and paratesticular tumours: a single centre experience over a 25-year-period. Klin Padiatr.

