# Supplementary material for: Trends of testis-sparing surgery for pediatric testicular tumors in South China
Source: BMC Surg. 2017 Mar 27;17:31. doi: 10.1186/s12893-017-0230-1 (PMC5369203; doi:10.1186/s12893-017-0230-1)
Supplement: Additional file 1: — Clinical characters of children with testicular benign tumors. (PDF 24 kb) [file 12893_2017_230_MOESM1_ESM.pdf]

Age	size	lateral	center	period	surgery	AFP
1	3.0	2	1	2	0	1
1	8.0	1	1	2	0	1
1	2.7	1	2	1	0	0
1	3.0	1	2	1	0	0
1	2.3	2	2	1	0	0
1	2.5	2	1	2	0	1
1	4.5	2	2	1	0	0
1	1.7	2	2	1	0	0
1	3.5	2	2	1	0	0
1	4.0	2	1	2	0	0
1	6.0	2	2	1	0	0
1	1.5	1	2	1	0	1
1	3.0	2	1	2	0	0
1	2.6	2	1	2	0	0
1	5.0	1	2	1	0	0
1	2.0	1	2	1	0	0
1	1.3	1	1	1	0	0
1	2.8	1	2	1	0	0
1	5.4	2	1	2	0	1
1	1.8	1	2	1	0	0
1	1.9	2	2	1	0	0
2	1.8	1	2	1	0	0
2	2.2	2	1	2	0	0
2	1.3	2	1	2	0	0
2	2.5	1	2	1	0	0
2	2.5	1	1	2	0	0
2	2.6	1	2	1	0	0
2	2.8	2	2	1	0	0
2	3.0	1	1	2	0	0
2	3.2	1	1	2	0	0
2	4.0	2	2	1	0	0
1	.5	1	2	2	1	0
1	2.0	2	1	2	1	1
1	1.3	1	2	1	1	0
1	2.0	2	1	1	1	0
1	.3	2	1	2	1	0
1	1.7	1	1	2	1	0
1	1.1	1	1	2	1	0
1	1.0	1	1	1	1	0
1	2.0	2	1	2	1	0
2	1.0	2	1	1	1	0
2	1.5	1	1	1	1	0
2	1.0	3	1	2	1	0

2	1.8	3	1	2	1	0
2	1.0	2	1	2	1	0
2	2.4	3	2	2	1	0
